# Heterogeneous early illness courses of Korean patients with bipolar disorders: replication of the staging model

**DOI:** 10.1186/s12888-022-04318-y

**Published:** 2022-11-04

**Authors:** Yejin Lee, Dongbin Lee, Hyewon Jung, Yunji Cho, Ji Hyun Baek, Kyung Sue Hong

**Affiliations:** 1 Department of Psychiatry, Samsung Medical Center, Sungkyunkwan University School of Medicine, 81 Irwon-ro, Gangnam-gu, 06351 Seoul, Korea; 2 Department of Digital Health, Samsung Advanced Institute for Health Sciences and Technology (SAIHST), Sungkyunkwan University, Samsung Medical Center, Seoul, Korea; 3 Samsung Biomedical Research Institute, Seoul, Korea; 4 Department of Psychiatry, University of British Columbia and Lions Gate Hospital, Vancouver, BC, Canada

**Keywords:** Bipolar disorder, Mood disorders, Staging, Staging models, Comorbidity

## Abstract

**Background:**

Clinical staging of bipolar disorder (BD) requires application of real-world data, as the next step in hypothesis. This study used the staging model to analyze the long-term course of BD in Korean patients based on clinical features and treatment responses to map the progression of bipolar illness from its early phase after the onset of illness.

**Methods:**

A total of 136 patients diagnosed with BD-I (*n =* 62) or BD-II (*n =* 74) were recruited. Their progressive stages were retrospectively evaluated. A multi-state model was used to calculate the probability of progression to each stage. Hazard ratios of covariates expected to influence different courses of BD were calculated. Using the Alda score, long-term responses to mood stabilizers depending on the current stage were compared.

**Results:**

Several sub-populations showed varied courses during the first five years after the onset of illness, with 41.5% remaining in stage 2 and 53% progressing to higher stages with shortened time for transition. Profiles of patients with BD-I and BD-II were different, suggesting biologically distinct groups. Comorbid psychiatric disorders, such as obsessive-compulsive disorder (OCD) and bulimia nervosa (BN) were associated with a recurrent course (stage 3a or 3b) or a malignant course (stage 3c or 4). Early age of onset, shorter duration of illness, older age at the start of medication, and poor response to lithium affected the illness progression.

**Conclusion:**

We were able to apply the stage model based on episode recurrence patterns in early illness courses of Korean patients with BD. The stage progression pattern differed from the early phase in BD-I and BD-II patients. Psychotic comorbidity, age at onset, age at starting psychiatric treatment showed associations with the illness progression.

## Background

Bipolar disorder (BD) is a chronic psychiatric disorder characterized by symptom recurrence and remission during patients’ lifetime after the onset of the illness. Life-long treatment is inevitable in BD to prevent recurrence. Long-term treatment response and recurrence patterns vary depending on the patient. The complexity of the illness hinders prediction of long-term prognosis.

A recent staging model shows the potential to understand the course of complex illness such as BD based on a unified model. McGorry et al. [1] have proposed the staging of mental disorders with an aim to develop a transdiagnostic model. Berk et al. [2] have adapted the staging model to BD which is largely defined by the occurrence and recurrence of mood episodes. Kapczinski et al. [3] have proposed an alternative model based on inter-episodic functional impairment and potential biomarkers. Duffy et al. [4] have developed a third model focusing on early stages of BD, before the onset of a full blown illness.

The staging model can facilitate our understanding of the course of a complex illness such as BD and identification of meaningful biomarkers that can be used in precision psychiatry.

Recent studies have reported the application of a staging model for analyzing the course of patients’ illness clinically [5, 6]. However, it is still unclear how the staging model can be used in clinical evaluation and decision-making process in BD. Previous studies have certain limitations. First, they did not show how the staging model can be used in conjunction with previous findings associated with long-term bipolar illness including comorbid psychiatric conditions. Second, previous studies mainly included biased populations and predominantly patients with BD-I and ethnically western populations. Third, they did not report whether the stage was associated with long-term treatment responses. Additional studies are needed to determine the clinical utility of the staging model in BD treatment.

In this study, we applied the staging model proposed by Berk et al. [2] in consideration of its applicability and usefulness to determine the course of long-term illness in Korean patients with BD. We have collected clinical data on illness courses of patients with BD including recurrence patterns, treatment changes and treatment responses. Using this database, we retrospectively evaluated patients’ long-term illness and applied the staging model based on the illness recurrence patterns. Next, we explored the overall stage progression pattern after the onset of illness (stage 2). We also sought to determine the clinical features associated with the pattern of BD progression. We also investigated whether patients’ current stages were associated with their responses to standard treatment.

## Methods

### Study participants

Patients who met the DSM-IV criteria for BD-I or BD-II and had received treatment at the Bipolar Disorder Clinic of the Samsung Medical Center, a tertiary-care university-affiliated hospital, were recruited between September 2019 and September 2021. Patients’ ages ranged from 18 years to 55 years. Those who had evidence of neurological disorders or general medical conditions related to mental symptoms were excluded. A total of 136 patients who met the above criteria and agreed to participate in the study were enrolled. The Institutional Review Board (IRB) of the Samsung Medical Center approved this study (IRB no. 2021-01-084). This study was conducted in accordance with the ethical standards of the relevant institutional committees and with the Helsinki Declaration.

### Clinical information

Clinical information were collected via direct interviews with patients, their available care-givers, and their physicians. Patients’ medical records were also used as a supplementary information source by psychiatrists (JHB, DL, YC and KSH) and a psychologist (HWJ). The Korean version of the Diagnostic Interview for Genetic Studies (DIGS) [8] was used to confirm patients’ diagnoses and disease history. All interviewers had at least two years of research experience using DIGS and participated in several consensus meetings in order to improve inter-rater reliability. Based on the accumulated information, patients’ diagnoses were re-established. The course of illness, symptom profiles, lifetime co-occurrence of other DSM-IV axis I disorders, and past history of suicide attempts were also evaluated.

### Retrospective assessment of progressive illness using the staging model

The evaluation based on a direct interview has been described previously [9–11]. Based on clinical information, study psychiatrists (JHB, DL, YC and KSH) and psychologists (HWJ and YL) with at least two years of clinical experience independently established the occurrence, duration, and temporal sequence of stages each month after patient’s initial symptom development. Since our primary interest was in early clinical course of BD, we assessed illness progression in the first five years after the onset of BD. The investigating psychiatrist and the clinician who saw each patient independently reviewed the information and arrived at a consensus regarding the pattern of stage progression. We also held regular consensus meetings to review the progression of each case.

In summary, the model consisted of five stages. Each stage was divided into four sub-stages (i.e., A, B, C, and D). Stage 0 was defined as increased risk of severe mood disorder with familial loadings. Stage 1 was characterized by mild or non-specific symptoms including impulsivity, irritability, and major depressive episode. The diagnosis of BD started with stage 2. BD was further differentiated into recurrent episodes based on depressive, hypomanic, and manic/mixed symptoms. Stage 3a was defined as recurrence of subsyndromal depressive or manic symptoms after the onset of BD. When depressive, hypomanic, or manic episode recurred but fully remitted for more than two months, it was considered as stage 3b. Stage 3c was characterized by incomplete remission of recurrent episodes with persistent residual or subthreshold mood symptoms. Lastly, stage 4 included multiple relapses of mood episodes without symptomatic or functional recovery for two years. When the first episode that qualified for onset of BD occurred and lasted for two years, it was considered as a chronic course with a fast transition from stage 2 to stage 4.

In general, patients enter higher stages as the disorder develops. However, after treatment, remission of symptoms may occur subsequently. To distinguish re-entry into a specific stage after the first entry, backward transition from stage 4 to stage 3 was defined as stage 3’ and stage 3c to stage b was defined as stage 3b’. In our model, subjects remained in the assigned stage after remission of the episode until transition to a consecutive stage.

### Assessment of clinical characteristics

Rated variables included age at onset, course of mood episodes, manifested symptoms, suicidality, and comorbid psychiatric conditions on a lifetime basis. Age of first exposure to mood stabilizer or atypical antipsychotics and age at onset were also explored. As age at first medication might differ from the age of BD onset, the duration of illness (DOI) was determined by subtracting the year of BD diagnosis from the current age at the enrollment.

Psychiatric comorbid conditions including anxiety disorder, obsessive compulsive disorder and alcohol use disorder were evaluated due to their associations with the long-term clinical course. We additionally evaluated borderline personality traits generally observed in BD [12] as these traits could affect the course of overall illness [13]. We used Personality Assessment Inventory-Borderline scale (PAI-BPD; [14]) to assess borderline personality characteristics in patients with BD. The PAI-BPD with 24 items was used to measure symptomatology of borderline personality disorder (M = 33.20, SD = 11.58). The scale included subscales to assess affective instability, identity problems, negative relationships, and self-harm [15].

### Assessment of response to long-term mood stabilizer treatment

We additionally compared the long-term effect of lithium depending on the patients’ current stage. All patients received standardized treatment prescribed by their treating physicians based on the standard treatment guideline for BD [16–19]. Two psychiatrists (JHB and KSH) independently reviewed the charts retrospectively and interviewed patients. The long-term treatment response to mood stabilizers was evaluated using the Alda scale [20]. The Alda scale comprises two subscales: (1) Alda A score, which evaluates the degree of improvement during the intervention, and (2) the Alda B score, which assesses confounding variables that affect the outcome leaving medication effect aside [21]. The total score is a composite score calculated by subtracting B from A.

### Statistical analyses

A multi-state model was used to analyze the relationship between the proposed stages of BD [22]. According to Markov assumption, the transition rate is independent of both the duration of remaining in the current state and the state visited prior to the current state [22]. The mstate package in R statistical software [23] was used to apply this model in order to represent all the proposed stages instead of treating such stages as covariates.

Hazard ratios of covariates involved in different courses of stage progression were calculated using the Cox proportional-hazards model. Covariates involved in progression from stage 2 to stage 3a or stage 3b and from stage 2 to stage 3c or stage 4 were analyzed. The following covariates were included: at least one parent with severe psychiatric illness, sex, working status, psychiatric comorbid conditions and cumulative scores of PAI-BPD. Due to variation in treatment history, we included age at onset, duration of illness (DOI), age first exposed to medication, and number of admissions to hospitalization in the analysis.

To compare responses to long-term mood stabilizers, we classified participants into three groups based on their current stage: early BD (stage 2; *n =* 64), recurrent group (including those in stage 3a and 3b; *n =* 31), and malignant course group (including those in stage 3c and 4; *n =* 41). Basic demographic characteristics of these groups were compared. Linear regression analyses were conducted after adjusting for covariates known to be associated with mood stabilizer responses, i.e., alcohol use disorder, personality disorders, and higher lifetime number of hospital admissions [24].

## Results

### Basic sociodemographic characteristics of participants

Sociodemographic and clinical characteristics of 136 patients included in the analysis are presented in Table 1. Sixty-two (45.6%) subjects met the criteria for BD- I and 74 (54.4%) subjects met the criteria for BD- II. The sample included both inpatients and outpatients. Forty (29.9%) subjects were never hospitalized. Forty-seven (35.1%) subjects were hospitalized once, while others (n = 47, 35.1%) were hospitalized twice or more.

**Table 1:** Sociodemographic and clinical characteristics of patients with BD (*N =* 136)

Characteristic	N (%)	Mean (SD)[range]
Gender		
Male	40 (29.4%)	
Female	96 (70.6%)	
Parental diagnosis of bipolar disorder	6 (4.4%)	
Marital status	34 (25.0%)	
Education level		
Middle school	2 (1.5%)	
High school	22 (16.2%)	
Some college	112 (82.4%)	
Working status, employed	96 (70.6%)	
Diagnosis		
BD-I	62 (45.6%)	
BD-II	74 (54.4%)	
Comorbidity		
Panic disorder	42 (31.1%)	
Alcohol-related disorders	14 (10.4%)	
Bulimia nervosa	12 (8.9%)	
GAD	11 (8.1%)	
OCD	9 (6.7%)	
Social phobia	9 (6.7%)	
Agoraphobia	8 (5.9%)	
Anorexia nervosa	2 (1.5%)	
Number of hospitalizations		
0	40 (29.9%)	
1	47 (35.1%)	
≥ 2	47 (35.1%)	
Age at onset, years		20.0 (7.0) [8.0–46.0]
Medication age, years		24.7 (7.4) [15.0–50.0]
Duration of illness, years		11.1 (7.7) [1.0–38.0]
PAI-BPD total score^a^		58.3 (14.0) [29.0–91.0]

*BD* Bipolar Disorder, *GAD* Generalized Anxiety Disorder, *OCD* Obsessive-Compulsive Disorder

^a^self-reported

The average age of onset for patients who were first assigned to stage 2 was 20.0 (SD: 7.0) years. First exposure to medication, mood stabilizer, or antipsychotics was delayed from disease onset in most cases, with a mean age of 24.7 (SD: 7.4) years. Table 1 lists psychiatric comorbidities, with panic disorder (31.1%) having the highest prevalence rate, followed alcohol-related disorders (10.4%) and generalized anxiety disorder (8.1%).

### Stage progression

Six (4.4%) patients reported familial loading of BD, which corresponded to the standard stage 0. The average duration spent at each stage was as follows: 6.6 (SD: 6.9) years in stage 1, 4.3 (SD: 6.5) years in stage 2, 0.9 (SD: 3.7) years in stage 3a, 0.2 (SD: 1.2) years in stage 3b, 0.7 (SD: 2.0) years in stage 3c, and 0.4 (SD: 1.7) years in stage 4. As patients who had already reached stage 2 were only included in our study, prevalence of familial bipolar disorder (stage 0) and duration of time spent in previous stage (stage 1) might not represent the common phenomenon of persisted subthreshold symptoms of BD [25].

**Fig. 1:**
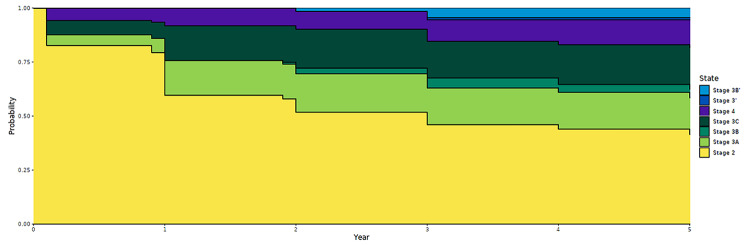
Probability of different stages in the first five years after onset of BD (*N =* 136)

Figure 1 shows the transition of subjects throughout the model in five years after the onset of BD. The horizontal axis shows years passed since the subjects’ entry to stage 2. The vertical axis shows cumulative probability of remaining in a certain stage. It was found that 41.5% of patients still remained in stage 2 and 40.3% of patients reached stage 3. In addition, 17.0%, 4.0%, and 19.3% of patients remained in stage 3a,stage 3b, and stage 3c, respectively, while 12.7% of patients advanced to stage 4. A total of 5.4% of patients who had reached stage 4 remained stable.

### Comparisons of stage progression between BD-I and BD-II

**Fig. 2:**
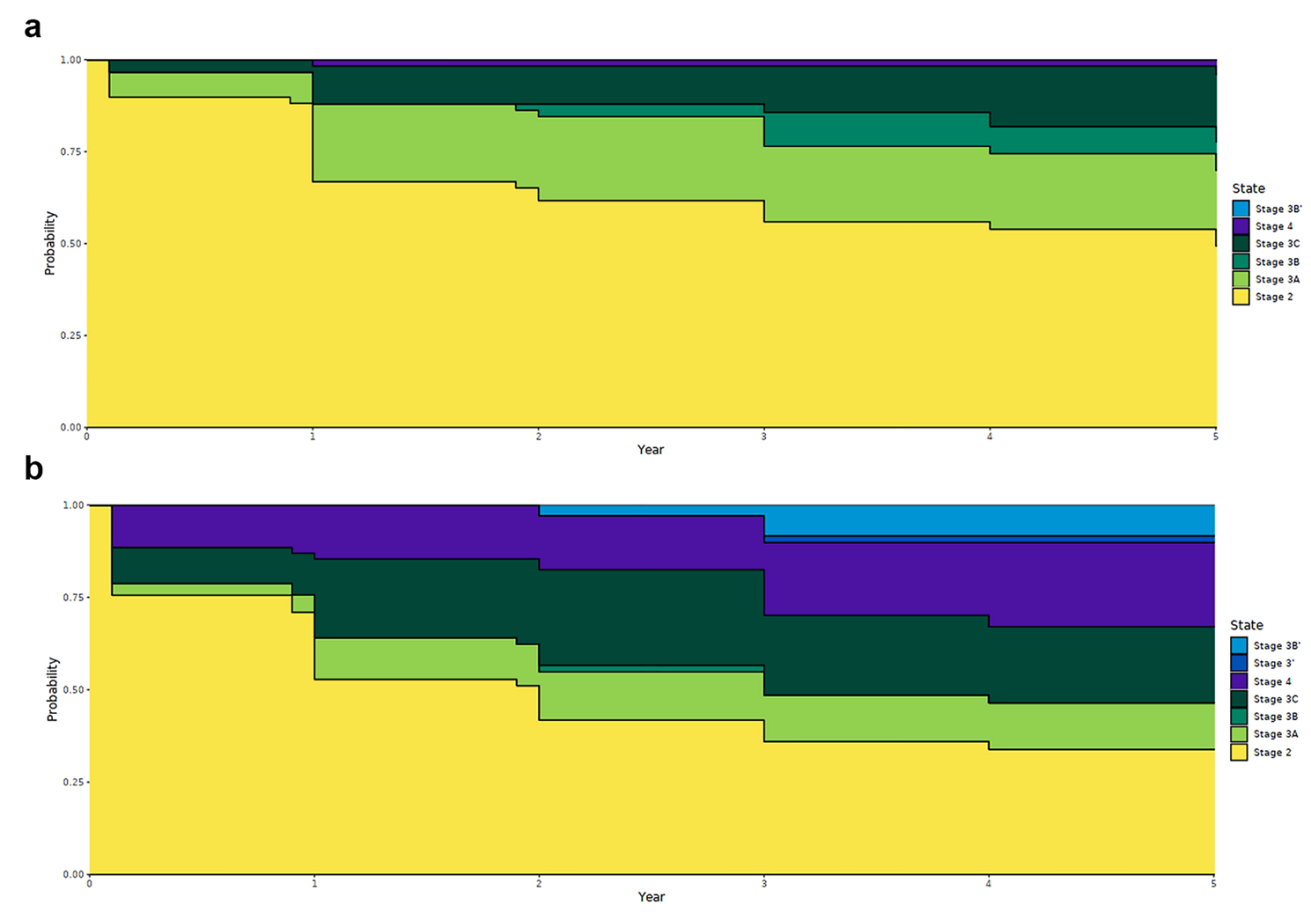
(A) Probability of different stages among patients with BD-I (*N =* 62), (B) Probability of different stages among patients with BD-II (*N =* 74)

Figure 2 A and 2B show transition of patients with BD-I (*n =* 62, 45.6%) and BD-II (*n =* 74, 54.4%) five years after enrolling in stage 2. The disease course of patients with BD-I (see Fig. 2 A) revealed that 49.4% remained in stage 2, while 20.5%, 7.8%, and 18.3% advanced to stage 3a, stage 3b, and stage 3c, respectively. Finally, 3.9% of the patients progressed to stage 4. Among patients diagnosed with BD-II, 33.9% remained in stage 2 (see Fig. 2B), while 12.6% and 20.6% reached stage 3a and stage 3c, respectively. The rate of patients who advanced to stage 4 was significantly higher in BD-II than in BD-I (22.7% in 3.9% in BD-II vs. 3.9% in BD-I, *p <* 0.01). In addition, 49.4% of patients with BD-I remained in stage 2, while 33.9% of those with BD-II remained in stage 2. However, their difference was not statistically significant (*p =* 0.07).

### Cox hazard regression models to determine the clinical factors associated with stage progression

**Fig. 3:**
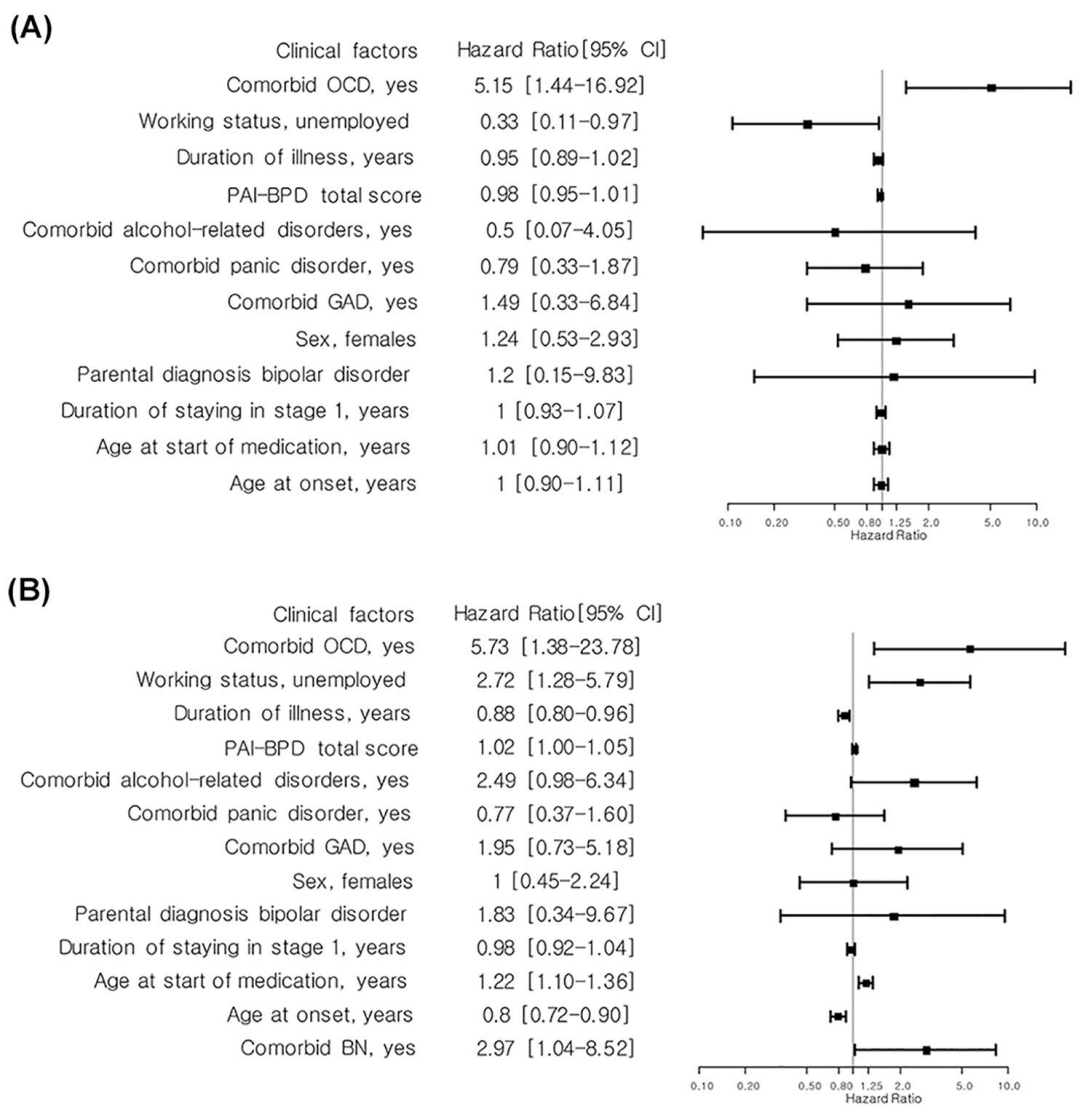
(A) Covariates that increased the risk of stage progression from stage 2 to stage 3a/3b, (B) Covariates that increased the risk of stage progression from stage 2 to stage 3c/4

The Cox hazard regression model was used to analyze several covariates associated with stage progression (see Fig. 3). The hazard ratio indicates the increase in transition rate for an added variable within a group [6].

The group with obsessive-compulsive disorder showed higher transition rate from stage 2 to stage 3a or stage 3b (HR = 5.15). Being unemployed also decreased the rate (HR = 0.33). Furthermore, we analyzed effects of such factors on disease progression from stage 2 to stage 3c or stage 4. Earlier age at onset, shorter DOI, older age at start of medication, and being unemployed increased the transition rate. Subjects with comorbid OCD and bulimia nervosa had higher risk of progression to stage 3c (HR = 5.73) or stage 4 (HR = 2.97). However, there was no difference in rate of comorbid psychiatric disorders between patients who remained in stage 2 and those who transited to higher stages. There was no significant difference in the transition rate to higher stages related to the duration of years in prodromal state (stage 1).

### Comparisons of clinical characteristics and long-term mood stabilizer responses depending on stages

We additionally classified participants into three groups depending on the current stage: early BD group (those in stage 2, *n =* 64), recurrent group (those in stages 3a and 3b, *n =* 31), and malignant course group (those in stages 3c and 4, *n =* 41). There were no significant differences in current age, age at onset, DOI, age first exposed to mood stabilizer or atypical antipsychotics, or psychiatric comorbid conditions among groups.

Of all participants included in this study, 57 subjects were treated with lithium, while 57 were treated with valproate. Significant differences were observed in Lithium Alda A (early BD group: mean = 7.39, standard deviation (SD) = 1.34; recurrent BD group: mean = 6.21, SD = 1.63; malignant course group: mean = 6.78, SD = 1.49; F = 4.10, *p =* 0.022; post-hoc analysis using Tukey’s method: early BD vs. recurrent BD, *p =* 0.031; recurrent BD vs. malignant BD, *p =* 0.776, early BD vs. malignant BD, *p =* 0.078) and total scores among groups (early BD group: mean = 4.35, SD = 2.15; recurrent group: mean = 2.07, SD = 2.50; malignant course group: mean = 2.74, SD = 1.79; F = 5.98, *p =* 0.004; post-hoc analysis using Tukey’s HSD : early BD vs. recurrent BD, *p =* 0.006; early BD vs. malignant BD, *p =* 0.032; recurrent BD vs. malignant BD, *p =* 0.620). These differences remained significant even after controlling for age, sex, comorbid alcohol use disorder and comorbid borderline personality traits (F = 4.81, *p =* 0.012 for Alda A score; F = 7.30, *p =* 0.002 for total score) [24].

## Discussion

In this study, we applied the staging model to Korean patients with BD based on comprehensive clinical information obtained from diverse sources including chart review and direct interviews with patients, their caregivers and treating physicians. This approach is both intuitive and applicable in clinical practice based on a series of symptoms in light of patient’s own clinical evolution [26].

Of several staging models, we applied the staging model proposed by Berk et al. [2]. The model proposed by Kapczinski et al. [3] also addresses clinically observed phases of illness progression. Although we used comprehensive clinical information, we did not have available measures validated to evaluate patients’ functional status and neurocognitive performance. Thus, we could not apply the model of Kapczinski et al. [3] to our data. Further study with sufficient markers to assess patients’ functional status is needed to apply Kapczinski et al. [3]’s model.

Stage progression patterns for several sub-populations in our study were distinct during the first five years after the onset of BD, especially for those with BD-I and BD-II. A prior study using the staging model [6] has included patients with BD-I only. Findings of our study are consistent with previous findings showing that BD-II is associated with a chronic illness course and more frequent depressive episodes [9, 27]. Such different stage progression patterns support the differentiation of BD-I and BD-II [28, 29]. In BD-II, a subset of patients reached stage 4 early in their course of illness, while others did not undergo relapse of episodes in 5 years. The interval (years) between episodes is widely distributed [30, 31], emphasizing heterogeneous features in the longitudinal course of BD-II.

The duration of transition was shortened when patients reached higher stages. In our study, the number of years spent in later stages 3 and 4 was less than a year. This transition was faster in later stages than in a previous study [6], while the progression in earlier stages (1 and 2) was even slower in the present study. Salvatore et al. [32] have clustered hetereotypic risk factors into early, intermediate, and late (prodromal) phases and found that the mean latency among phases is reduced gradually. The mean latency was 4.7 ± 6.9 years between early and intermediate antecedent phases. It was 8.4 ± 14.4 weeks between first-episode symptoms and syndrome. The acceleration also suggests that early manifest BD and malignant groups have different illness course, highlighting the need for considering current stage in planning treatment strategies.

Notably, several studies have shown different rates of stage progression patterns reflecting the progression of illness during five years after the onset of BD, a time frame for early intervention of the targeted population. In a previous study by van der Markt et al. [6], 85% of subjects experienced stage progression in five years, with 7% remaining at the same stage. The rate of stage progression in our study was lower than that in the previous study (53% in our study versus 85% in the study by van der Markt et al., *p <* 0.0001), while a substantial number of patients stayed in stage 2 in our study (41.5% vs. 7%, *p <* 0.0001). Because our study subjects were recruited from a BD clinic, timely and appropriate treatment might have delayed the progression of illness. These findings suggest the need for using a staging model in clinical practice as a prophylactic intervention against recurrent episodes.

Treatment can alter the disease course. Early onset and delayed medication were significant factors leading to chronic course of BD in our study, in line with a previous study involving subjects with cycle acceleration [33]. In a study using the staging model based on recurrent episodes, earlier age at onset and treatment with fewer psychotropic medications during patients’ lifetime were associated with higher stages [34]. Joslyn et al. [35] have also found that early age of onset is associated with factors that can negatively impact long-term outcomes. Chronic BD spent shorter time in higher stages than in stage 2 possibly due to its rapid transition. The association between gender and an increased risk of stage progression reported by van der Markt et al. [6] was not replicated in our study. Identification of individual factors for personalized care requires assessment and adjustment of clinical interventions.

Other elements should be considered as they represent stage-specific markers [1, 26]. Previous studies have included mood symptoms such as prodromal subsyndromal depressive or manic symptoms or specific bipolar onset [6]. In our study, we included psychiatric comorbid conditions as covariates associated with long-term bipolar illness [36]. As expected, patients with comorbid OCD, alcohol-related disorders, or bulimia nervosa showed increased rate of transition to higher stages. Due to a small sample size of those with comorbid OCD (*n =* 9), it was difficult to conclude the role of comorbid OCD in the clinical course of BD. However, a previous study have generally shown that comorbid OCD is associated with worse clinical courses in BD [37]. Furthermore, we identified distinct factors associated with recurrent BD (stage 3a/3b) or malignant course (stage 3c/4). Recurrent BD is associated with better prognosis as it includes remission of episodes, whereas malignant BD is characterized by residual state without complete remission, although these two courses have recurrent episodes in common [38]. An interesting finding of our study was that unemployment increased the risk of malignant course, but lowered the risk of episodes following remission. We speculate that severe impairment or loss of function might have occurred in individuals later in the course of established BD. However, the state of being unemployed, which indicates the current work situation, may not suggest inability to work. Kapczinski et al. [39] have included psychosocial functioning as an index of illness progression.

The staging model not only enhances our understanding of BD, but also sheds light on treatments with differential value across stages. Previous studies have reported mixed results of treatment response. A study by Berk et al. [40] has pooled 12 BD studies and found that patients in the earliest phases of the illness have more favorable responses to treatment. However, staging was not a significant factor in antidepressant response in a randomized trial [41]. Staging did not moderate the randomized treatment effect of lithium vs. quetiapine [34]. Our study showed that current stage was associated with long-term mood stabilization in response to lithium therapy. The discrepancy in results might be attributed to different samples. In our study, both average duration of illness and mean age were nearly 10 years earlier than those in previous studies. Our sample consisted of a diverse range of patients with early to chronic courses of BD. Application of staging model in treatment decisions and prognosis requires determination of stage-specific pharmacological treatment.

The neuroprogression model could explain underlying pathophysiological mechanisms of the staging model of BD. Namely, recurrent episode could cause deficient neurogenesis, increase cell shrinkage and apoptosis, and compromise neuronal function and structure, eventually leading to worse treatment responses and increased vulnerability to relapse and chronicity [42]. Progressive structural brain changes were observed in patients with recurrent episode compared to patients with the first episode. Diverse neurobiological mechanisms including epigenetics, telomere shortening, inflammation, oxidative stress and mitochondrial dysfunction might be involved in this process [40].

The present study had strengths in that it used real world clinical data actually observed by treating physicians repeatedly. We not only obtained data from patients’ recall or electronic health records, but also held regular consensus meetings to improve the reliability of stage definition for enhanced conceptualization and measurement of staging [43].

Our study has several limitations. First, the small sample size might have affected our study findings. Our dataset was divided into BD-I (*n =* 62) and BD-II (*n =* 74) to compare the probability of stage progression. This inevitably caused estimation problems due to the small sample size. Kupfer [44] has emphasized that cultural context should be considered to develop a satisfactory model, adding value to our study. To the best of our knowledge, no prior study has applied staging models to an Asian population. Second, there could be a recall bias regarding patients’ early illness. Comorbid psychiatric disorders in this study were rated in their lifetime without reflecting different patterns of comorbidity or illness trajectories (e.g., timing of onset of all comorbidities experienced) [45]. We speculate that clinical features that preceded the onset of bipolar disorder at stage 1 could be addressed by Berk et al. [2]’s model. In addition, we tried to re-formulate patients’ course of illness using all available information sources and repeated contacts with patients themselves, their caregivers and their treating physicians. Third, all patients were recruited and treated at a BD specialized clinic, making it difficult to generalize our study findings. Fourth, we only applied the staging model based on recurrent episodes. A possible restriction of our study was that clinical recovery was distinct from the concept of psychosocial functioning [46]. Although our clinical data included the status of employment, they lacked varied aspects to assess inter-episodic functioning. Markt et al. [7] have mentioned that psychosocial functioning might be rated differently depending on cultural background. As we discussed variables associated with illness progression, whether they could be applied to the model of Kapczinski [3] should be addressed further. Descriptions of clinical stages of BD still need operationalization or further refinements from current consensus on terminology [42]. For instance, whether a mixed episode meets the criteria for a certain stage can be disputed, leading to systemic error.

Despite these limitations in mind, this study demonstrated the feasibility of applying the staging model based on real-world data involving Korean patients with BD. Our findings suggest that clinical staging can be used to integrate diverse courses of BD. Exacerbation of BD and resistance to treatment provide insight into illness progression at a group level ranging from early to recurrent and chronic conditions. Known variables that could aggravate the prognosis were confirmed. Additional variables and biomarkers are reflected in this framework.

## Conclusion

The present study provided additional evidence that distinct courses of BD can appear in five years after the onset of BD. In addition, the stage progression pattern differed between BD-I and BD-II patients from the early phase. Psychiatric history of comorbid symptoms, resistance to treatment aggravated prognosis, and further studies are needed to expand the clinical staging to map the progression pattern of BD.

## Data Availability

The dataset that supports the findings of this study are available from the corresponding author upon reasonable request.

## Abbreviations

BD: Bipolar disorder
BD-I: Bipolar I disorder
BD‐II: Bipolar II disorder
OCD: Obsessive-compulsive Disorder
DIGS: Diagnostic Interview for Genetic Studies
DOI: Duration of illness
PAI-BPD: Personality Assessment Inventory-Borderline scale
GAD: Generalized Anxiety Disorder
BN: Bulimia Nervosa.
